# Shape-Setting of Self-Expanding Nickel–Titanium Laser-Cut and Wire-Braided Stents to Introduce a Helical Ridge

**DOI:** 10.1007/s13239-024-00717-2

**Published:** 2024-02-05

**Authors:** Martina Bernini, Rudolf Hellmuth, Mike O’Sullivan, Craig Dunlop, Ciara G. McKenna, Agnese Lucchetti, Thomas Gries, William Ronan, Ted J. Vaughan

**Affiliations:** 1 Biomechanics Research Centre (BioMEC), School of Engineering and Informatics, University of Galway, Galway, Ireland; 2 Vascular Flow Technologies, Dundee, UK; 3 Division of Imaging and Science Technology, School of Medicine, Dundee, UK; 4 National Heart and Lung Institute, Imperial College London, London, UK; 5 Institut für Textiltechnik of RWTH, Aachen University, Aachen, Germany

**Keywords:** Peripheral stent, Nickel–titanium, Heat treatment, Shape-setting, Finite element analysis, Helical flow

## Abstract

**Purpose:**

Altered hemodynamics caused by the presence of an endovascular device may undermine the success of peripheral stenting procedures. Flow-enhanced stent designs are under investigation to recover physiological blood flow patterns in the treated artery and reduce long-term complications. However, flow-enhanced designs require the development of customised manufacturing processes that consider the complex behaviour of Nickel-Titanium (Ni-Ti). While the manufacturing routes of traditional self-expanding Ni–Ti stents are well-established, the process to introduce alternative stent designs is rarely reported in the literature, with much of this information (especially related to shape-setting step) being commercially sensitive and not reaching the public domain, as yet.

**Methods:**

A reliable manufacturing method was developed and improved to induce a helical ridge onto laser-cut and wire-braided Nickel–Titanium self-expanding stents. The process consisted of fastening the stent into a custom-built fixture that provided the helical shape, which was followed by a shape-setting in air furnace and rapid quenching in cold water. The parameters employed for the shape-setting in air furnace were thoroughly explored, and their effects assessed in terms of the mechanical performance of the device, material transformation temperatures and surface finishing.

**Results:**

Both stents were successfully imparted with a helical ridge and the optimal heat treatment parameters combination was found. The settings of 500 °C/30 min provided mechanical properties comparable with the original design, and transformation temperatures suitable for stenting applications (*A*_f_ = 23.5 °C). Microscopy analysis confirmed that the manufacturing process did not alter the surface finishing. Deliverability testing showed the helical device could be loaded onto a catheter delivery system and deployed with full recovery of the expanded helical configuration.

**Conclusion:**

This demonstrates the feasibility of an additional heat treatment regime to allow for helical shape-setting of laser-cut and wire-braided devices that may be applied to further designs.

**Supplementary Information:**

The online version contains supplementary material available at 10.1007/s13239-024-00717-2.

## Introduction

**Fig. 1:**
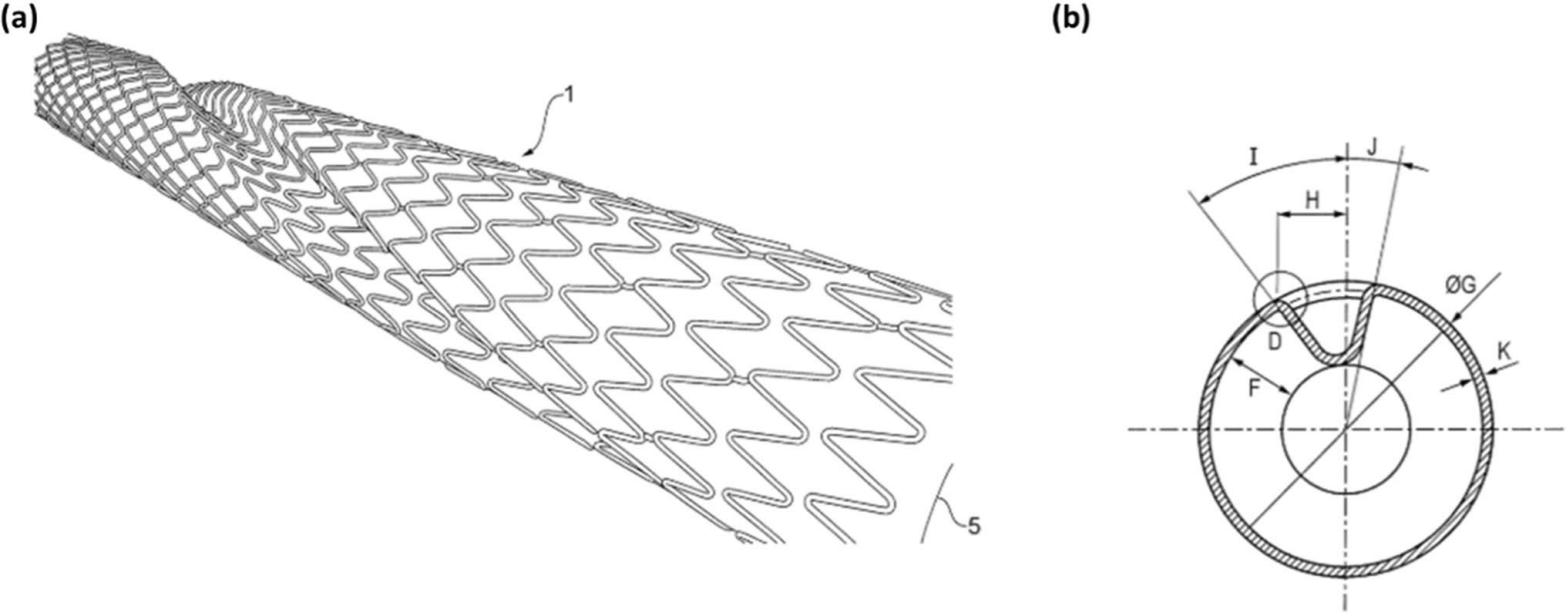
Perspective view of a stent having a helical ridge (**a**), and schematic view of the cross-section (**b**) [15]

Atherosclerosis in the peripheral arteries is commonly treated with the implantation of self-expanding Nickel–Titanium (Ni–Ti) stents [1, 2]. Nevertheless, the outcomes of this procedure are affected by complications in the long-term, with a reduction of vessel patency of 80% in the iliac artery, and as low as 30% in the infra-popliteal segments at 2-year follow-up after treatment [3]. One of the primary contributors to failure following stent implantation is the local alteration of hemodynamics both in the implanted region and in the downstream non-stented segment. A physiological hemodynamic flow is characterised by the presence of helical flow patterns [4, 5], which has been found to play an important role in the stabilisation of flow and the minimisation of disturbances [4]. However, stented segments [6] are associated to the presence of areas with flow separation, recirculation, and regions of low time-averaged wall shear stress [7], which influences the endothelialisation process and creates a pro-atherogenic environment [4]. This suggesting that stents might concur to the development of several complications as in-stent restenosis (ISR) and thrombosis [5, 6, 8, 9], with wire-braided and laser-cut devices having their own unique effects [8]. With this in mind, several commercial endovascular devices [10, 11] that aim to enhance the local hemodynamics in treated peripheral arteries were developed. Among these, the Spiral Laminar Flow™ (SLF™) prosthetics grafts (Vascular Flow Technologies Ltd, Dundee, United Kingdom) contain a helical inducer running along the distal end of the graft that allows the recovery of a helical flow. The efficacy of the graft was proved in a clinical trial where the device was implanted in patients who needed above-the-knee and below-the-knee bypass and demonstrated improved patency rates of respectively 81% and 57% at 24 months [10]. Also, the BioMimics 3D® Vascular Stent (Veryan Medical Ltd., Horsham, United Kingdom) exploits the swirling centreline of the device to impose a helical geometry on the artery to produce the desired helical blood flow. The stent reported positive safety and effectiveness, with freedom from loss of primary patency of 70% at 24 months [11]. In this study, a particular attention is given to the SLF™ technology, which has received CE mark and FDA approval. This technology, first applied to prosthetic grafts for the treatment of peripheral artery disease (PAD), has provided encouraging outcomes in animal and clinical studies [12–14], which has led to a translation to PAD Ni–Ti stents. The SLF™ stent is intended to have a cylindrical outside geometry where a part of the stent struts form a spiral internal ridge, and to potentially be covered with polytetrafluoroethylene (ePTFE) [15]. The ridge profile and the helix angle (Fig. 1) are optimised for a good compromise between helical flow development and energy loss due to laminar friction.

To produce such flow-enhanced stent designs, custom manufacturing processes need to be developed that carefully consider the complex behaviour of Ni–Ti. This particular alloy exists in different crystalline phases, whereby a transformation from the austenite phase to stress-induced martensite occurs when a load is applied to the material at a temperature above the austenite finish temperature (*A*_f_). An additional phase (R-phase) featuring a rhombohedral distorted martensite is recognised, which competes with the more stable martensite for the succession of austenite thanks to lower barriers to formation. These transformations produce two phenomena, the superelasticity property and the shape-memory effect. The superelasticity property is controlled by stress: above the transition temperature, deformations of the material cause a stress-induced phase change, which allows for elastic recovery up to 8–10% of strain once the load is removed. The shape memory effect is controlled by temperature: the material can be deformed easily at low temperatures (up to 8–10% strain), and recover the initial shape when the temperature is raised above *A*_f_. Controlling the *A*_f_ temperature of the alloy within the range of 15–25 °C [16] enables superelastic behaviour at body temperature (37 °C), which is a fundamental property for peripheral stent applications as it allows severe deformation to be accommodated during limb movement [17]. Generally, the starting point for stent fabrication is the wire or tubing that can be used to produce wire-braided or laser-cut frames, respectively. Ni–Ti wires are inter-woven to produce braided stents, while tubes are laser-cut to obtain the desired stent design and followed by post-manufacturing processes such as chemical etching, mechanical deburring, shape-setting, sand blasting and electro-polishing [18]. During the manufacturing process, both wire-braided and laser-cut stents require a heat treatment step to establish the final shape-set and to ensure optimal mechanical and thermal properties within the superelastic window. Recently, attention as grown on alternative manufacturing process that enables the fabrication of complex and functional designs that could otherwise not be realised with conventional techniques [19], this including additive manufacturing of Ni–Ti stents [20].

It is well documented that the mechanical and physical properties of Ni–Ti alloy are the result of the manufacturing process, and in particular of the thermomechanical treatments that essentially control the transformation temperatures [21], mechanical properties and surface layer finishing [22]. The heat treatment is normally performed using an air furnace or a salt bath, and it is followed by a rapid quenching in cold water [23]. The effects of the manufacturing process on Ni–Ti material properties are well-reported, for wire specimens in particular [23–26] or for *ad hoc* designed coupon with characteristic strut dimensions [27]. Pelton *et al.* [24] investigated the effects of cold working and heat treatment on Ni–Ti wires for medical applications and found that heat treatments between 350 and 450 °C resulted in substantial increase of *A*_f_ and a decrease of the loading plateau stress. Heat treatments above 500 °C resulted in smaller increases of *A*_f_, with a drastic reduction in the loading plateau stress observed at 550 °C. Interestingly, the ultimate tensile stress (UTS) increased with heat treatment in the range 300–450 °C, but dramatically decreased above 550 °C. These conclusions found support in a following study by Liu *et al.* [25] where wires were heat treated in comparable conditions and phase transformation temperatures and mechanical properties assessed. Results were used to inform the shape-setting of a unit of an aortic aneurysm stent, which provided a stable configuration when subjected to shape-set in a furnace at temperatures of 500 °C and 550 °C for a period longer than 10 min, with temperature being the most crucial factor to obtain the desired shape. Also Marchand *et al.* [23] investigated the effects of two successive non-identical shape-setting on wire-braided stents for heart valve implantation. Physical and mechanical tests addressed *A*_f_ temperature, elastic modulus, elastic limit stress, elastic recoil, and different combinations of subsequent thermal treatments on Ni–Ti wires with the same heating history. The authors found an increase in effective initial stiffness with temperature, while a decrease in limit stress occurred with increasing time and duration of the process. Overall, the literature shows that transformation temperatures and mechanical properties are influenced by heat treatment and cannot be manipulated independently, providing additional challenges in the manufacturing process. However, there is a lack of knowledge on the influence of heat treatments and the mechanical properties changes at the device-level, for instance in terms of crush resistance and radial behaviour which are relevant for stent application [28].

In this study, a manufacturing process for introducing a helical inner ridge inspired by the SLF™ graft technology was developed and trialled on both laser-cut and wire-braided stents. First, the geometry of the custom-built fixture used to induce the helical shape was improved through computational analysis and then a study on the more suitable heat setting parameters was conducted. The effects of the shape-setting process on the mechanical performance, transformation temperature and functionality performance of the helical device were assessed and compared to the reference stent design. Mechanical performance of the device was investigated with radial compression and parallel plate crush testing. The post-treatment transformation temperatures were assessed to guarantee the device working in the superelastic range at body temperature. The bending and deliverability of the helical devices was addressed by observing their kinking behaviour and by loading them onto a delivery system to perform *in vitro* deployment in a straight mock vessel. Once optimised, this manufacturing process could be used to modify the Ni–Ti mesh of cardiovascular devices to induce spiral flow [29] and could potentially be applied to different stent designs.

## Materials and Methods

### Self-Expanding Ni–Ti Stent Frames

**Fig. 2:**
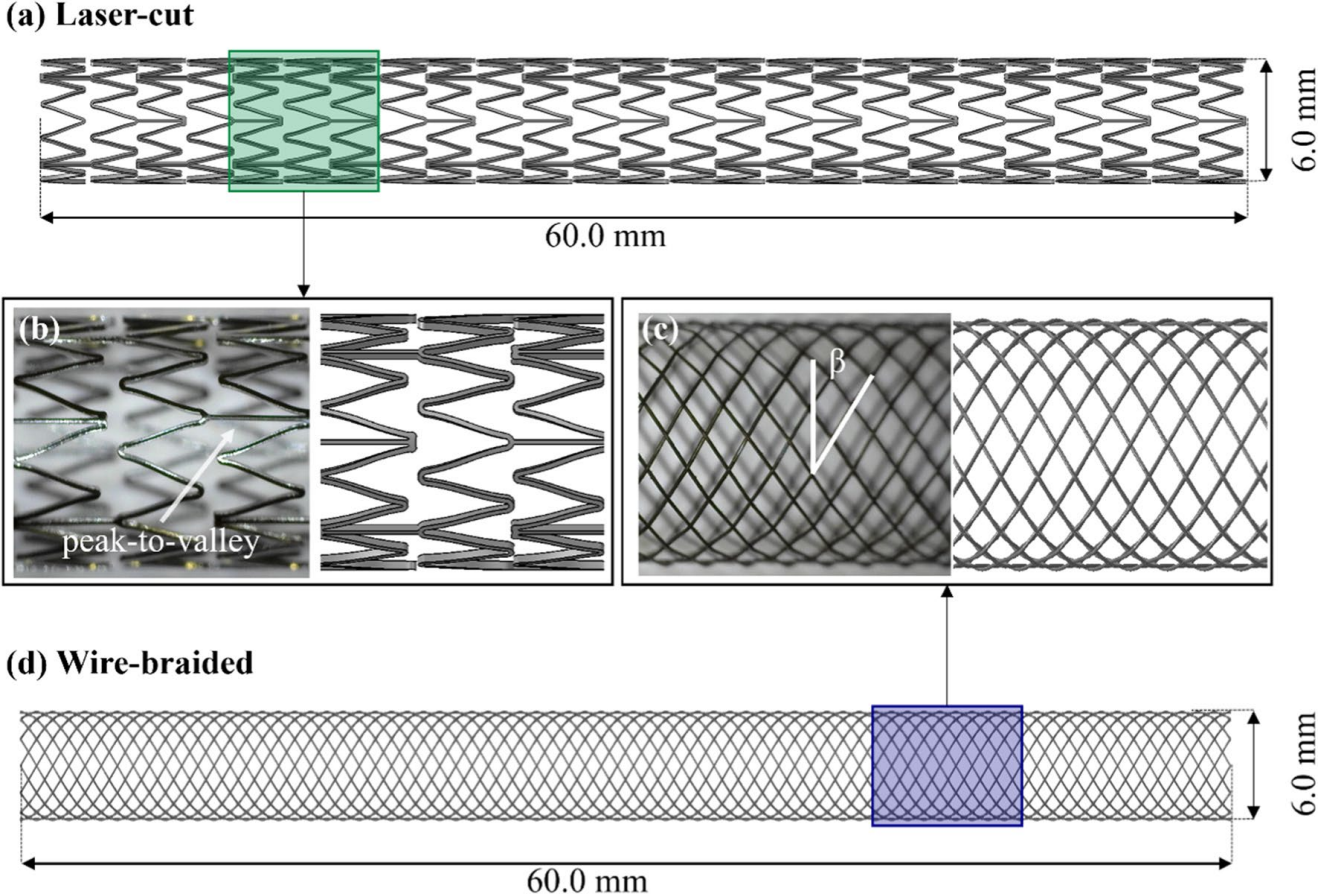
Stent frames: laser-cut stent (**a**, **b**), and wire-braided stent (**c**, **d**)

Laser-cut and wire-braided frames featuring a nominal diameter of 6.0 mm and a length of 60.0 mm were used in this study (Fig. 2a, d). The laser-cut stents were inspired by the self-expanding Zilver PTX (Cook Medical, USA) device, which has an open-cell Z-shaped design in a peak-to-valley arrangement and features a strut thickness of 200 μm (Fig. 2b). These stents were initially manufactured by ADMEDES GmbH (Pforzheim, Germany), through a process consisting of laser-cutting from a 2 mm diameter Ni–Ti tube, followed by an expansion to the final nominal diameter of 6.0 mm in a two-step heat treatment. The wire-braided stents were fabricated by the Institut für Textiltechnik of RWTH Aachen University (Aachen, Germany) from Ni-Ti wires (Fort Wayne Metals, Indiana, USA) with a diameter of 100 μm, such that the crossing point offered a thickness comparable to the laser-cut device (~200 μm). The braiding consisted of 24 wires woven into a 1-over-1-under pattern and featuring a braiding angle β = 30° (defined in Fig. 1c), which is comparable to commercial devices [30, 31]. The alloy composition and starting properties of the tubing and wire were not made available to the authors.

### Process for the Introduction of the Helical-Ridge

The helical ridge was introduced on both stent types through a manufacturing process that consisted in fastening the stent in a custom-built fixture to provide the desired shape, before placing it in an air furnace for shape-setting. Initially, the stainless steel fixture (Fig. 4a) was used for providing the shape deformation, whereby the stent was placed on the female mandrel and deformed to the helical shape by positioning the male parts at a temperature of 10 °C. Then, the assembly of the stent and the fixture was placed in an air furnace for a heat treatment cycle (Carbolite CWF Laboratory Chamber Furnaces, Gero, Neuhausen, Germany), followed by rapid quenching in cold water.

#### Improvement of the Fixture Geometry for Helical Deformation

**Fig. 3:**
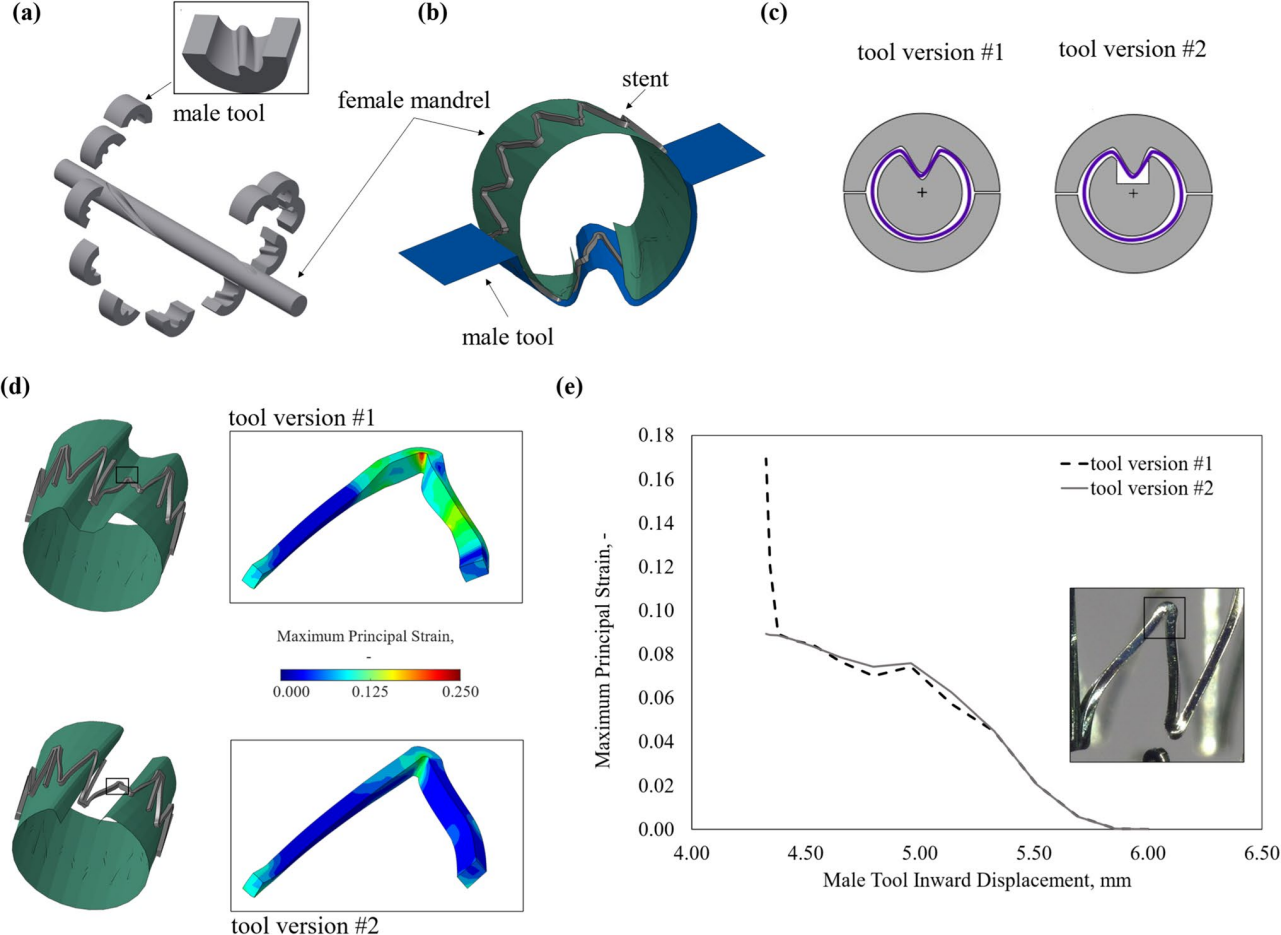
Tool optimisation with finite element analysis: **a** expanded view of the female and male tools that compose the fixture, **b** subunit of the stent and rigid surfaces of the custom-built fixture as modelled in the FEA, **c** schematic of tool versions, **d** comparison of stent configurations post shape deformation with details of maximum principal strain in the strut peak, and **e** trend of maximum principal strain in the strut during the displacement of the male tool

A batch of the laser-cut stents was dedicated to preliminary feasibility tests, which aimed at improving the procedure to induce the shape deformation. The geometric design of the custom-built tool was improved through a finite element analysis (FEA) with ABAQUS/Explicit software (v. 2017, SIMULIA, Dassault Systèmes). A sub-unit of the laser-cut device was modelled and discretised with 37,920 hexahedral elements with reduced integration (C3D8R). Ni–Ti material parameters derived from the calibration process performed in a previous work [32, 33] were used, with temperature dependency parameters (*∂σ*/*∂T*) taken from literature [34]. The custom-built tool was modelled with two rigid surfaces (male and female parts), whose interaction with the stent was defined using the general contact algorithm, assuming a friction coefficient of 0.1 (Fig. 3b). A temperature of 10 °C was considered for the analysis, consistently with the real procedure. Two versions of the tool were compared: (i) the first utilised a female mandrel with a cut profile matching the male counterparts, (ii) whereas the second had a more rectangular shape to remove the contact between male/female parts (Fig. 3c). The maximum principal strain induced at the peak of the strut was monitored during the shape deformation process (i.e., displacement of the male part). The comparison between tool #1 and #2 showed a similar final shape, although tool #1 induced an average strain in the strut peak of over 0.164 due to the tight constrain imposed by the mechanical contact between female and male parts. Contrastingly, the re-design of tool #2 reduced the peak strain to 0.089 (Fig. 3d, e), while maintaining the desired shape, and was therefore chosen to perform the manufacturing process in this study.

#### Improvement of Shape-Setting Step

A heat treatment performed in air furnace was performed to achieve the shape-setting of the helical stents. Six combinations of processing temperature (*T*_p_) and time (*t*_p_) were considered, as reported in Table 1. Firstly, the effects of temperature were investigated by keeping a fixed time of *t*_p_ = 50 min and changing the temperature *T*_p_ = 400, 450, 500 °C. Secondly, the effect of a fixed temperature *T*_p_ = 500 °C was investigated with varying processing time of *t*_p_ = 30, 40, 50 min. Three stents (*n* = 3) were produced for each combination of parameters both for the laser-cut and wire-braided frames.

**Table 1:** Combinations of heat treatment settings

Effect of processing temperature			
* T*_p_ (°C)	400	450	500
* t*_p_ (min)	50	50	50
Effect of processing time			
* T*_p_ (°C)	500	500	500
* t*_p_ (min)	30	40	50

### Mechanical and Physical Characterization of the Helical Ridge Stents

To evaluate the functional performance of the helical stents, devices were subjected to parallel plate crush and radial compression testing. Material characterisation testing was carried out using differential scanning calorimetry (DSC) and scanning electron microscopy (SEM) to measure the transformation temperatures between austenite and martensite phases and compare the surface finishing. Finally, the deliverability of the devices was also evaluated in terms of their ability to withstand bending, be loaded onto a catheter delivery system and deployed in a synthetic mock vessel. All tests were compared with a control (either laser-cut or braided), without the helical technology.

#### Crush Testing with Parallel Plates

**Fig. 4:**
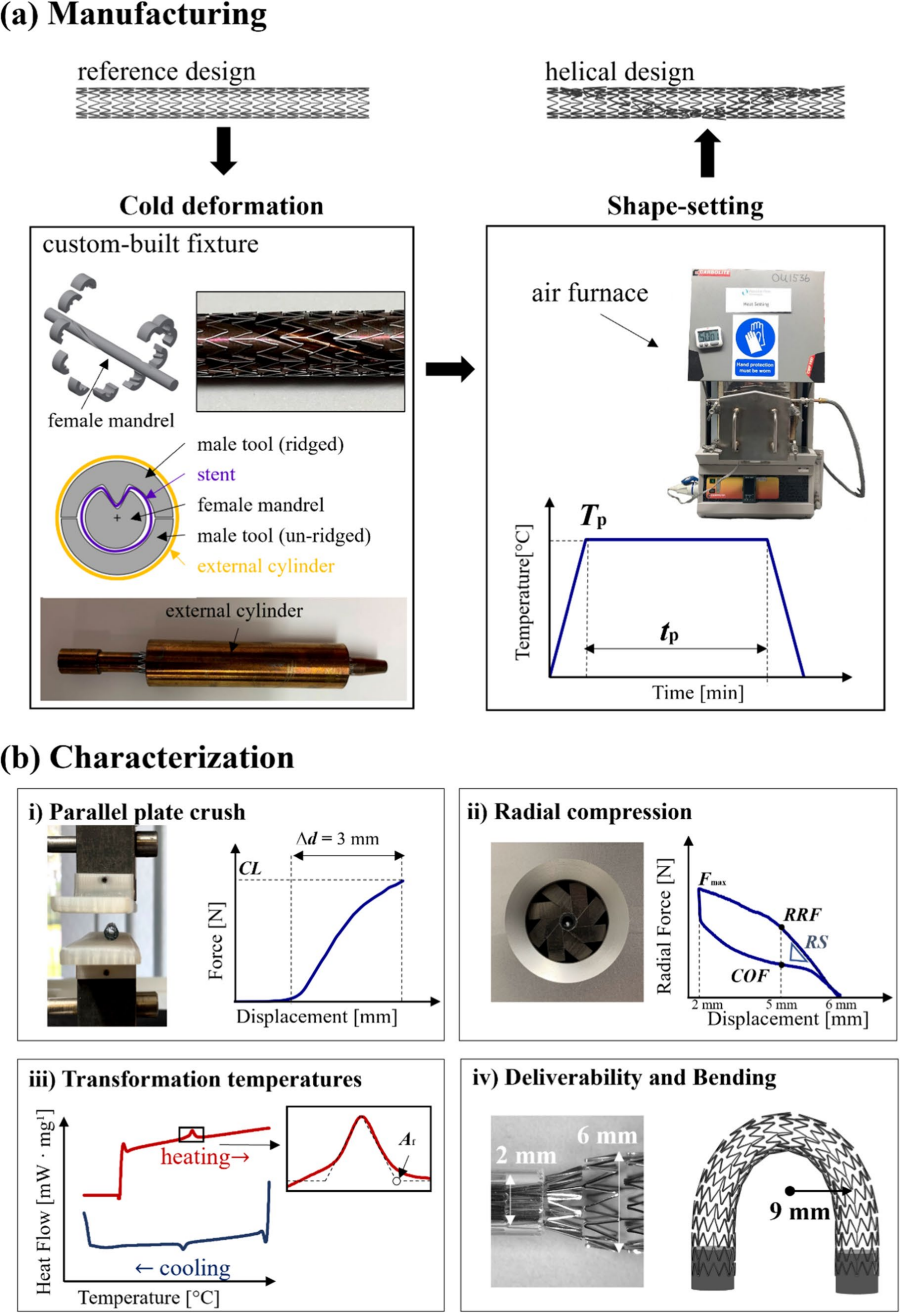
Schematic of manufacturing process (**a**) and characterization tests (**b**)

Crush testing was performed with the LP Plus Tensile Test Machine (Ametek Lloyd Instruments Ltd., United Kingdom) equipped with a 50 N load cell (900 Series, Tailored Test Solutions Ltd, United Kingdom) and custom-made parallel plate grips (Fig. 4b-i). A control on crosshead displacement was applied to compress the stent at least to half of the nominal diameter (*Δd* = 3 mm) and the corresponding force at the load cell was recorded as the crush load (*CL*) [28]. Testing was carried out in a water bath to ensure a temperature of 37 °C and stents were permitted to stretch in the axial direction.

#### Radial Compression Testing

Radial compression testing was performed with an 8-plate crimping head (RCM-H60, MPT Europe) mounted on a uniaxial test machine (Zwick Roell, GmbH & Co., Germany) that was equipped with a 100 N load cell (Fig. 4b-ii). The stents were crimped at a displacement rate of 0.1 mm s^−1^ from their full-unloaded configuration to a diameter of 2 mm followed by uncrimping without applying any constraint to the axial stretching of the stent. The plates were at 37 °C and the friction generated between the plates was subtracted to the radial force recorded, as explained in previous works [30, 32, 35]. This test assessed the radial strength (*RS*), defined as the slope up to the radial resistive force (*RRF*), the force at maximum crimping (*F*_max_) and the chronic outward force (*COF*) [30, 36]. The *RRF* and *COF* represent the forces respectively on the loading and unloading portions of the curve (see schematic in Fig. 4b-ii), when a deployment in a reference vessel of 5 mm diameter is considered [37].

#### Transformation Temperatures

To assess the influence of processing temperature and time during shape-setting, differential scanning calorimetry (Netzsch DSC 214 Polyma, Gerätebau GmbH, Germany) was employed [38]. Samples of 6.2 ± 0.2 mg were cut from the struts and wires and sealed in pierced aluminium pans before undergoing a heating step (−40 °C to + 100 °C), followed by a cooling step (+100 °C to − 80 °C) at a rate of 10 °C min^−1^. Three samples were tested for each manufacturing condition and transformation temperatures were evaluated as shown in Fig. 4b-iii. Surface finish was investigated with scanning electron microscopy (SEM S-4700, Hitachi) under an acceleration voltage of 15 kV.

#### Deliverability and Kink Test

Crimping and bending tests assessed the potential effects of the helical ridge on these deformation modes (see Fig. 4b-iv). First, the stents were loaded onto a commercial catheter delivery system and deployed into a silicone mock vessel (Dynatek Labs Inc., Galena, MO, USA) that featured inner diameter of 5 mm. Then, the stents were enforced to bend to a radius of curvature of 9 mm to assess the kinking behaviour [30]. This radius of curvature was chosen as representative of the worst-case deformation that could occur *in vivo* for above-the-knee regions in the femoropopliteal artery segment [39].

### Statistical Analysis

The statistical analyses were performed using Prism (v.9.4.1, GraphPad Software, San Diego, CA, USA). The Welch version of the one-way ANOVA test was employed to compare the equality of means between groups, and it was followed by Dunnett’s T3 multiple comparisons test to evaluate each heat treatment combination against the reference design with a statistical significance of p ≤ 0.05.

## Results

### Investigation on the Most Suitable Heat Treatment Parameters

**Fig. 5:**
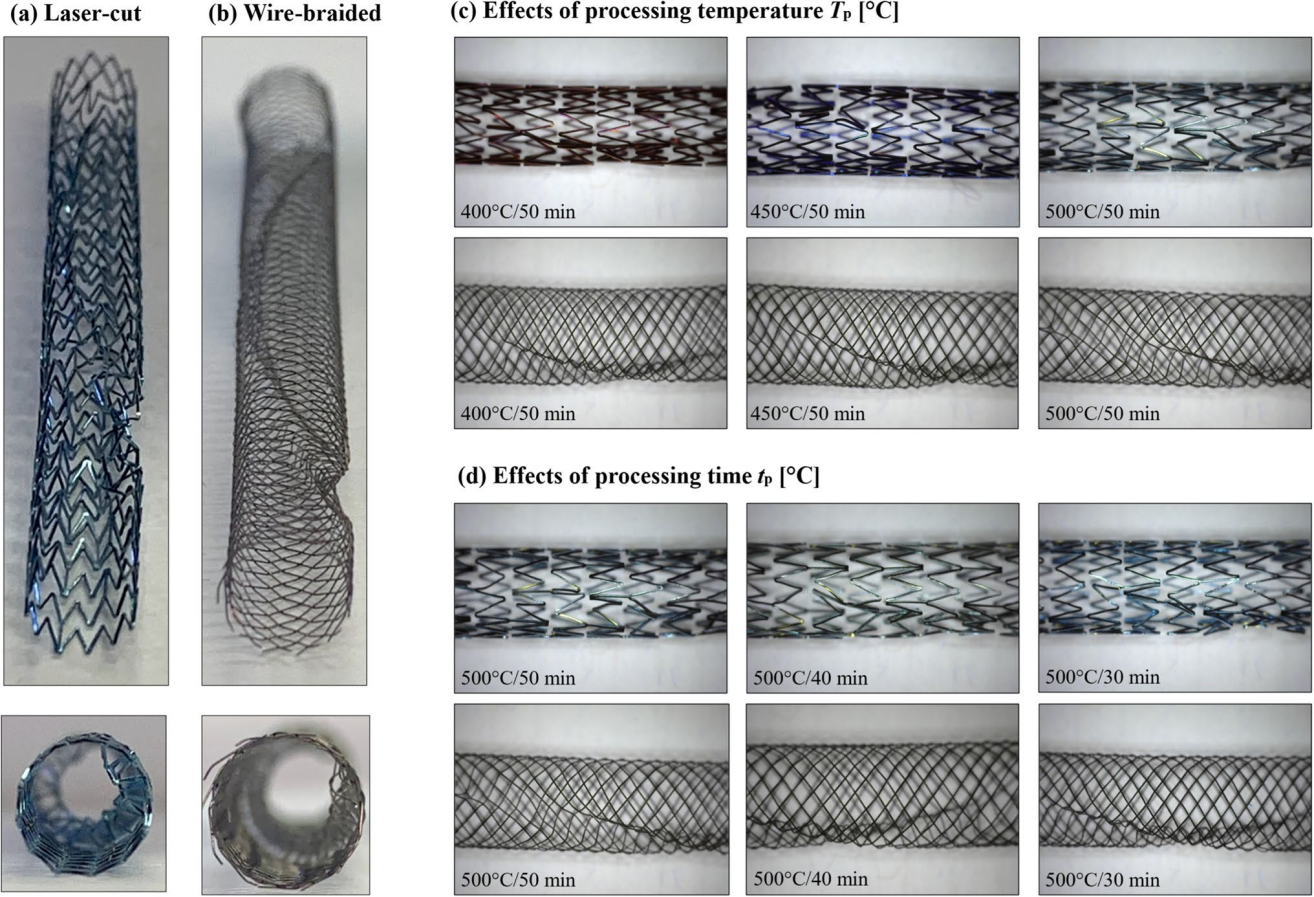
Helical design produced on the laser-cut frame (**a**) and the wire-braided frame (**b**): comparison of the outcomes of different heat setting parameters (**c**, **d**)

The helical ridge was successfully manufactured on both laser-cut and wire-braided stents, with no visual differences across the heat treatment groups found. Some variation of the surface was observed on the laser-cut frame (Fig. 5) where the stents processed at higher temperatures and shorter times showed a bright shiny metallic appearance, likely associated with a thin oxide layer, whereas stents displaying a dark appearance were processed at lower temperatures and longer times and are likely associated with a thicker oxide layer [1].

#### Effects on the Crush Test with Parallel Plates

**Fig. 6:**
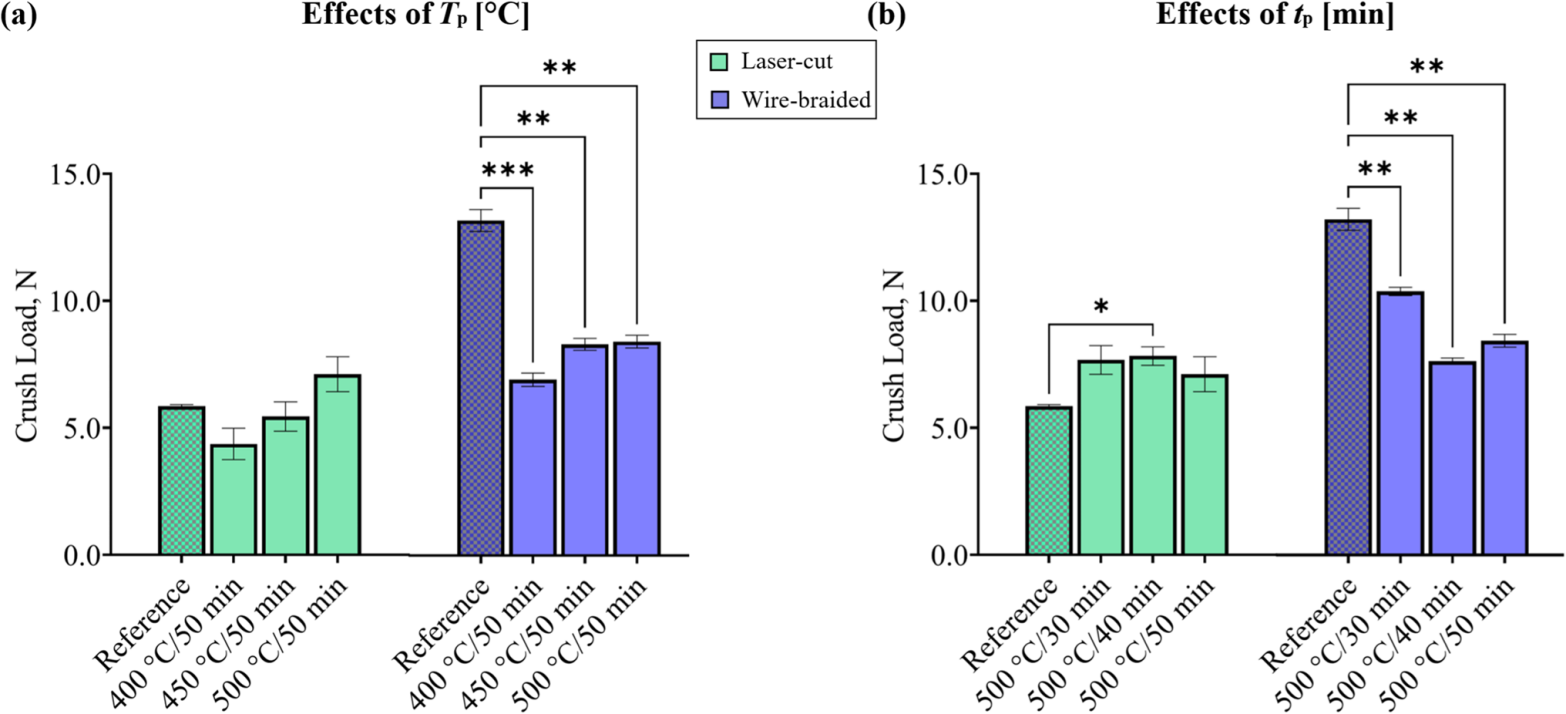
Crush load with parallel plates: **a** effects of processing temperature, and **b** effects of processing time. Welch test and Dunnett’s multiple comparison test compared the average *CL*; ns: p > 0.05, *p ≤ 0.05, **p ≤ 0.01, ***p ≤ 0.001, ****p ≤ 0.0001

The reference stent recorded a crush load (*CL,* as indicated in Fig. 4b-i) of 5.9 N and 13.2 N for laser-cut and wire-braided stents respectively (Fig. 6). Significant differences within stent frames were found, with the laser-cut design being more influenced by the processing time rather than by temperature, while the braided frame was significantly influenced by both temperature and time. When comparing each heat treatment to the reference, the laser-cut design showed a significant increase in *CL* (+33.7%) for the combination setting with a *T*_p_ = 500 °C and *t*_p_ = 40 min. The braided design showed a significant reduction in the *CL* for all the heat treatment combinations compared to the reference, with the largest drop (−47.5%) caused by the lowest temperature T_p_ = 400 °C and longest time *t*_p_ = 50 min. Supplementary Material is reported in the Appendix.

#### Effects on the Radial Behaviour

**Fig. 7:**
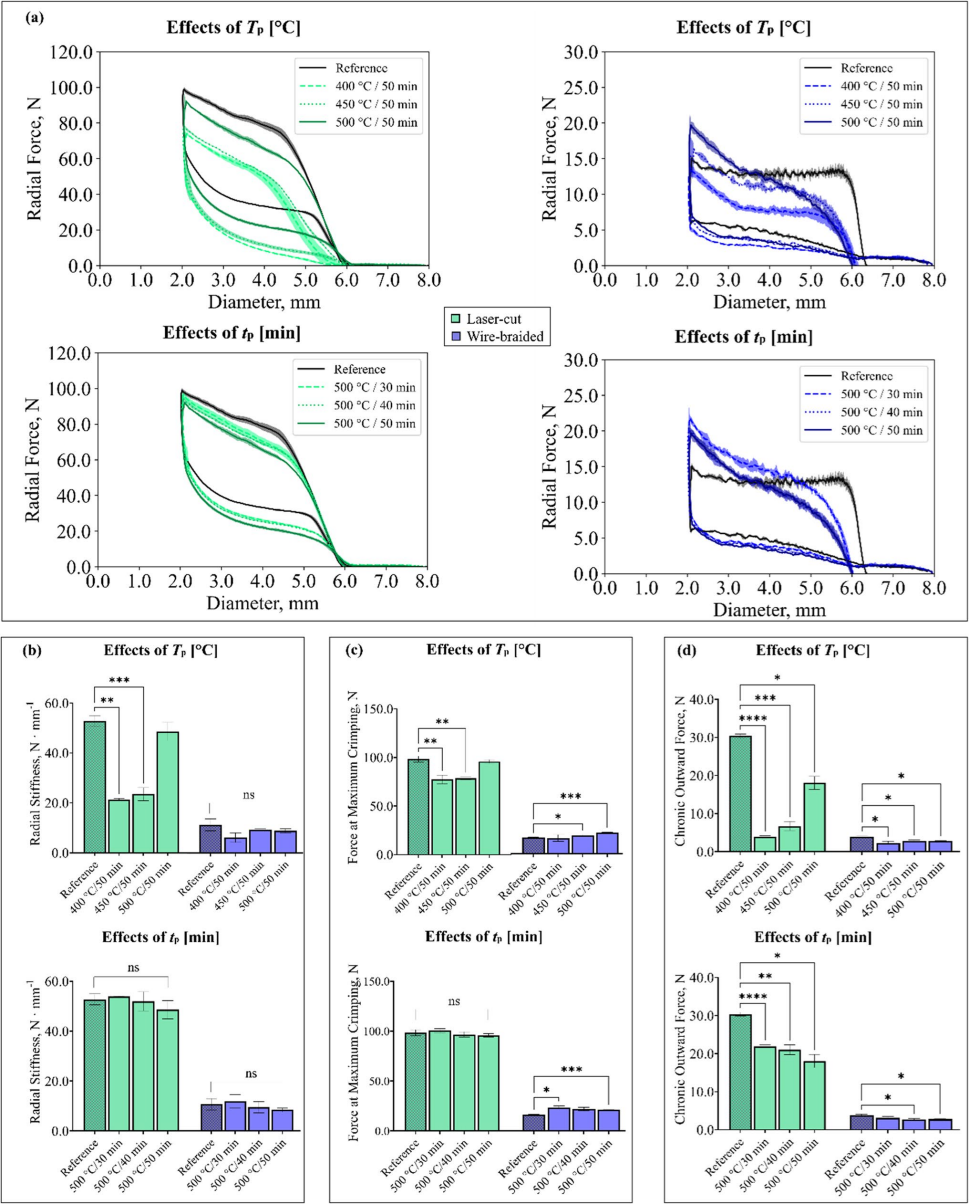
Radial force curves for laser-cut and wire-braided stents, showing the effects of processing temperature and time (**a**); statistical analysis of the effects of *T*_*p*_ and *t*_*p*_ on radial stiffness (**b**), force at maximum crimping (**c**), and chronic outward force (**d**). Welch test and Dunnett’s multiple comparison test  were performed; ns: p > 0.05, *p ≤ 0.05, **p ≤ 0.01, ***p ≤ 0.001, ****p ≤ 0.0001

Radial force curves are shown in Fig. 7a, where the effects of processing time and temperature are decoupled and compared with the reference design. The radial stiffness (*RS*), the maximum force at crimping (*F*_max_), and the chronic outward force (*COF*) are reported in Fig. 7b–d.

Overall, the heat treatment caused a decrease in radial performance with similar trends identified for both laser-cut and wire-braided stents. The largest drop in the radial curve was found by reducing processing temperature, even though it was mitigated by shortening the processing time.

In the laser-cut stents, reducing the processing temperatures caused a significant decrease in all the radial parameters compared to the reference, with major effects on *COF* (−87.0%, − 77.9%, − 40.5% at *T*_p_ = 400 °C, 450 °C and 500 °C, respectively). Otherwise, the processing time also had significant effects on *COF* (−27.8%, − 30.8%, − 40.5% at *t*_p_ = 30 min, 40 min and 50 min, respectively)*.* In the wire-braided stents, the *COF* was significantly impacted by processing temperature (−40.5%, − 28.8%, − 27.7% at *T*_p_ = 400 °C, 450 °C and 500 °C, respectively) and processing time (−18.8%, − 30.5%, − 27.7% at *t*_p_ = 30 min, 40 min and 50 min respectively). The processing time had a significant impact also on *F*_*max*_ (+45.5%, + 34.7%, + 31.3% at *t*_p_ = 30 min, 40 min and 50 min respectively), while no effects were found on *RS*. Supplementary Material is reported in the Appendix.

#### Effects on the Transformation Temperatures

**Fig. 8:**
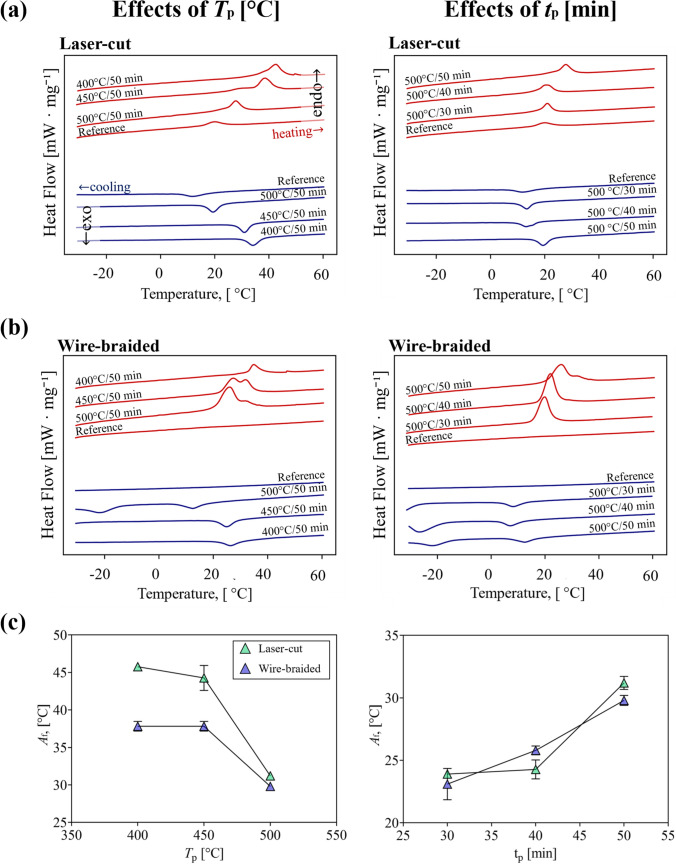
Heat flow curve showing the effects of processing temperature and time on **a** laser-cut and **b** wire-braided stents; **c** austenite finish (*A*_*f*_) temperatures trends plotted against the processing temperature *T*_p_ (left) and the processing time *t*_p_ (right)

The austenite finishing temperature (*A*_f_) was recorded from the heat flow curves during the heating and step of DSC testing (Fig. 8a, b). The transformation temperature of the reference laser-cut frame was *A*_f_ = 24.3 °C, while no peak was found within the tested range of temperatures for the reference wire-braided frame. The trend of *A*_f_ following the heat treatment at different processing temperature and time is shown in Fig. 8c. A similar trend was highlighted for laser-cut and wire-braided frames, with *A*_f_ showing a nonlinear decrease for rising processing temperature (*T*_p_) and a nonlinear increase for rising processing time (*t*_p_). Supplementary Material is reported in the Appendix.

#### Effects on the Surface Finishing and Composition

**Fig. 9:**
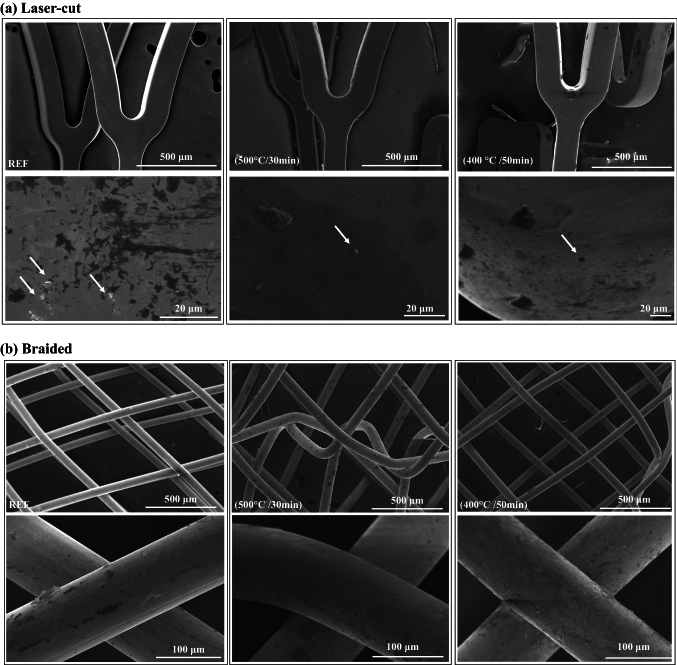
SEM imaging of laser-cut (**a**) and wire-braided (**b**): the left column shows the images of the reference stent (REF), the mid column shows the images of the most suitable manufacturing settings (500 °C/30 min), while the right column shows the images of the most unsuitable manufacturing settings (400 °C/50 min). For each combination of manufacturing settings, two figures (top and a bottom) are reported, which represent the same sample at two different magnifications

The comparison of SEM images revealed a similar surface appearance between the reference device and the helical stents that underwent the helical shape-setting process (Fig. 9). Some defects, namely dark and light spots, were found in all samples including the reference ones, probably owing to oxide and carbide inclusions generated in previous manufacturing steps (e.g., melt process) [40].

### Results of Deliverability and Kinking Tests

**Fig. 10:**
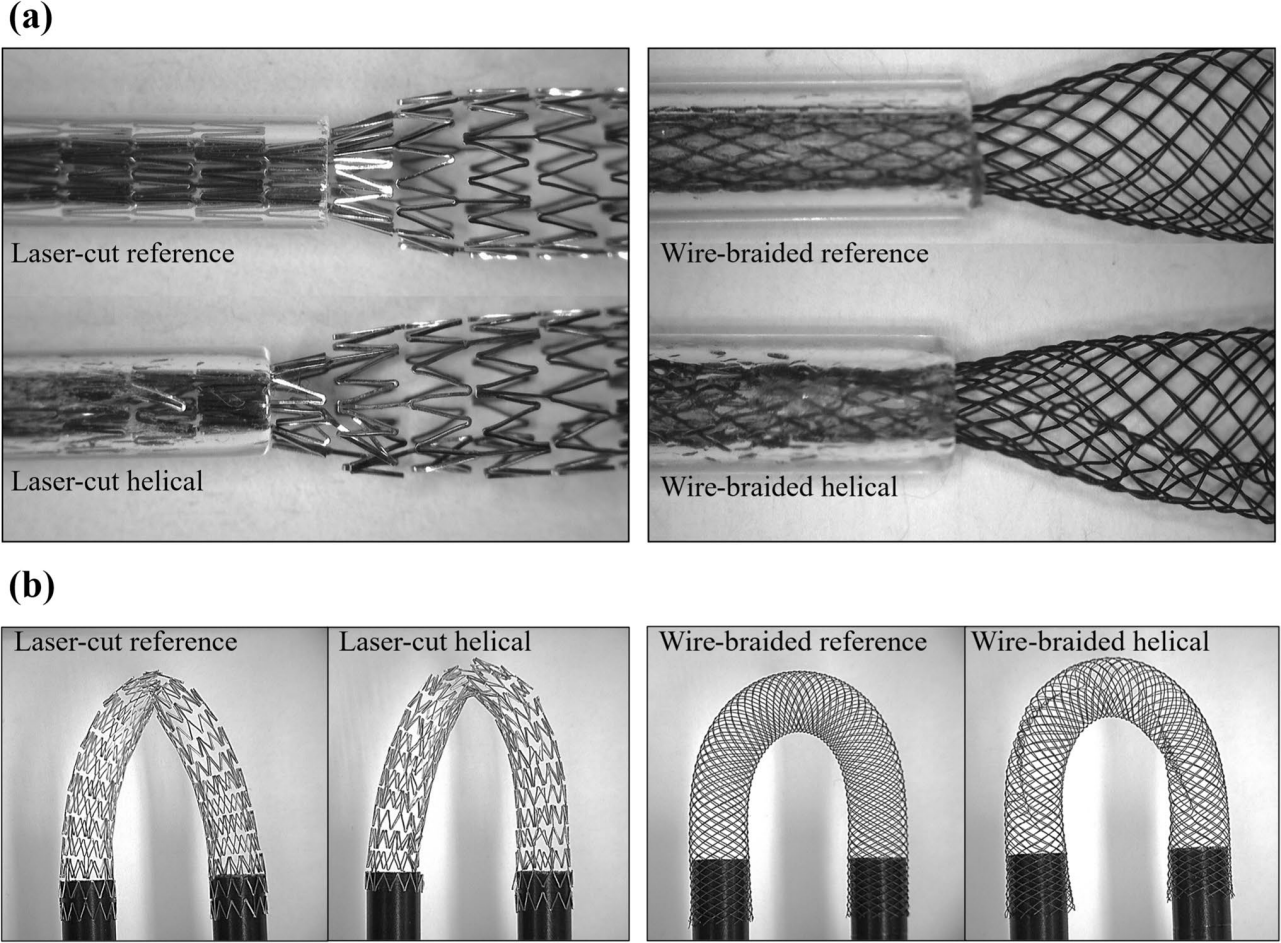
Reference and helical design (resulting from the most suitable combination of shape setting parameters, 500 °C/30 min) compared in the deliverability test (**a**) and kink test (**b**)

A qualitative comparison of reference and helical stents (manufactured with the most suitable shape setting parameters) under crimping and re-expansion suggested no differences (Fig. 10a). Both for laser-cut and wire-braided frames, the helical stent was successfully loaded onto a catheter delivery system and deployed in a straight mock vessel, recovering the desired helical shape in the expanded configuration. This outlined that, despite the presence of the inner ridge, no interference with the inner catheter was found and stents could be implanted through a minimally invasive procedure. Bending profiles (Fig. 10b) showed a slight kink in both reference and helical laser-cut stents, while wire-braided frames showed superior behaviour under kink deformations (the cross-sectional area profile was maintained). These results confirmed that the addition of the helical ridge did not impact the deliverability and kinking performance of the devices.

## Discussion

In this study, a Ni–Ti stent for the treatment of PAD with a potential flow-enhanced design was presented. In details, the work here described dealt with the manufacturing process required to introduce a helical ridge on both laser-cut and wire-braided self-expanding Ni–Ti stents. The process consisted of a shape-setting, where the stent was first deformed into the desired shape with a custom-built fixture followed by a heat treatment in an air-furnace. Both the deformation tool and the shape-setting protocol were improved to allow the introduction of the helical inner ridge without compromising the mechanical performance or the deliverability of either laser-cut or wire-braided stents. A systematic investigation of the mechanical and thermal performance of the modified devices was carried out through parallel plate crushing, radial compression testing and differential scanning calorimetry. Low processing temperature (*T*_p_ = 400 °C, 450 °C) influenced the mechanical performance causing a significant drop the chronic outward force (*COF*), and altered the transformation temperatures, with the effects of increasing the *A*_f_ above body temperature. On the other side, shortening the processing time (*t*_p_) showed fewer effects on the mechanical properties and the transformation temperatures. The most suitable combination of heat treatment parameters (*T*_p_ = 500 °C, *t*_p_ = 30 min) was found, as it provided the minimum drop in the *COF* (−27.8% for laser-cut, − 18.8% for wire-braided) and austenite finish temperature compatible with stenting application (23.90 °C for laser-cut, 23.10 °C for wire-braided).

While the manufacturing routes of traditional self-expanding Ni–Ti stents are well-established, the process to produce alternative stent designs and configurations is rarely reported in the literature. Recently, the application of an inner helical inducer to a prosthetic graft has shown promising clinical outcomes in the medium-term [10], and it is believed that it could be applied to stents to enhance the blood flow in the treated arteries. To the authors’ knowledge, this study represents the first attempt to expand the use of an inner helical ridge on self-expanding Ni–Ti stents, both laser-cut and wire-braided classes, and directly addresses the mechanical and physical properties at the device level. The helical ridge shape deformation required individual stent struts to undergo severe amount of twisting at hinge regions during the forming process, which was quantified to be 6.8-fold higher than the during a regular expansion process of a stent. First, an FEA optimisation of the fixture for shape-setting was carried out to reduce the deformations in the critical regions to avoid fracture of devices (seen in earlier trails), then, a thorough exploration of the parameters for a heat treatment in an air furnace was performed.

The role of heat treatment on the material-level properties of Ni–Ti is well-reported for wire specimens in particular [24–26], however, there is a lack of knowledge on the effects on mechanical and functional performance at the device level. In this study, the heat treatment parameters were systematically explored, and it was found that these resulted in significant differences in mechanical functionalities, which required optimisation of its parameters, namely processing temperature *T*_p_ and time *t*_p_. Generally, lowering the temperature of the heat treatments (e.g., *T*_p_ = 400 °C) was not beneficial for the mechanical properties as it caused a significant decrease of the crush load resistance (reduction up to 25.4% for laser-cut, 47.5% for wire-braided) and the radial behaviour (reduction of *COF* up to 87.0% for laser-cut, and 40.5% for wire-braided). The processing temperature had also greater effects on the temperatures that dictate the transformation from martensite to austenite, as previously reported in other studies on Ni–Ti wires [24, 25]. The higher *T*_p_ the lower the austenite finish temperature *A*_f_. However, for *T*_p_ = 500 °C, an austenite finish temperature of *A*_f_ = 31.2 °C (laser-cut) and 29.8 °C (wire-braided) was recorded, which was too close to body temperature. For implant applications, it should ideally be between 15 < *A*_f_ < 25 °C [16]. With the decrease of the duration of the heat treatment to *t*_p_ = 30 min, the loss in mechanical performance could be mitigated for both devices and was also beneficial for tuning the *A*_f_ to a value well-below body temperature, compatible with stenting applications. Through SEM images, it was found that the shape deformation and following heat treatment process did not substantially alter the surface finish of these devices (Fig. 9).

Lastly, the deliverability of the helical stents was compared to the reference design. Stents were successfully loaded onto a sheath-catheter delivery system (typically with a diameter of approx. 2 mm [37]) and implanted in straight mock vessels. Different bending behaviour was observed between laser-cut and wire-braided devices, with the latter showing a superior resistance to kinking but more importantly, no difference was found with the introduction of the helical ridge. Both deliverability and kink tests assessed that the helical modification is compatible with stenting application. The modified design can attain and maintain the crimping diameter in the delivery system, undergo a severe bending and eventually recover the expanded helical configuration.

This study is not exempt from limitations. To manufacture devices under a larger range of processing temperatures (*T*_p_) and times (*t*_p_), a limited number of samples (*n* = 3) was produced for each combination. To ensure that the statistical result can be generalised to a larger population, a greater sample size would be required. It is also worth noting that the mechanical responses was here assessed through parallel crush and radial tests, while stents *in vivo* are usually subjected to several deformations simultaneously (e.g., torsion, bending and axial compression [41]) that might increase the risk of fracture. In the direction of addressing the behaviour under realistic conditions, a further study has investigated the level of deformation in a Thiel embalmed cadaveric model in the presence of either a reference or a helical stent, focusing on the distal superficial femoral artery under a various range of limb movements [42, 43]. This combined with computational modelling (e.g., FEA) will allow to assess the level of strain reached in the device under realistic loading conditions experienced *in vivo*. Additionally, the assessment of realistic loading conditions will serve as a basis to quantify the configuration and deformation of deployed devices to set up a proper fatigue assessment, as indicated by the ISO standard 5840 [44]. Undeniably, fatigue life is an important aspect of the performance of a Ni–Ti stent, therefore durability of pulsatile and non-radial motion which may arise from musculoskeletal activities shall be thoroughly investigated in the future for the development of this device. Concerning fatigue, while the shape-setting step in the helical stents has not impacted the smoothness of the surface and the roundness of edges priorly achieved (Fig. 9), a further step of electropolishing is recommended after the introduction of the helical ridge, as it would improve durability and corrosion resistance [26]. More importantly, the capability of the SLF™ design in inducing a spiral laminal flow was not addressed in this work, however, it’s potential has been shown with *in vitro* investigation, computational fluid dynamics simulation and swine pre-clinical model [45, 46]. While the development of spiral flow has been found in bare stents [45], future investigations through *in vitro* and computational analyses will examine the potentiality of a covered helical stent to better direct the blood flow [47]. Lastly, clinical studies will be required to investigate the safety and efficacy of the helical device. An important aspect to investigate concerns the orientation of the ridge around the vessel circumference once deployed to assess its interaction with a plaque/dissection. It is thought that the width of the spiral ridge (approx. < 15% compared to the stent diameter) combined with pre-dilation of the artery lumen and oversizing of the stent will avoid poor apposition, and this combined to an enhanced flow could help in decreasing restenosis.

## Conclusions

The aims of this paper were to present the development and improvement of a manufacturing method to introduce a flow-enhanced design in self-expanding Ni–Ti stents, which will serve as an inducer to recover the natural blood pattern. A reliable manufacturing method consisting of a sequence of shape deformation and heat treatment was developed and improved to induce a helical ridge onto laser-cut and wire-braided frames. A thorough exploration of heat treatment parameters revealed that the combination of 500 °C/30 min allowed a minimal change in the mechanical performance of the device and the thermal properties of the material, both compatible with stenting application. Overall, the study not only demonstrated the feasibility of the approach on both laser-cut and braided stents, but identified a set of most suitable manufacturing conditions that are applicable to both classes, with this suggesting the reliability of the methodology and its potential application to other stent designs.

### Supplementary Information

Below is the link to the electronic supplementary material.

Supplementary file1 (DOCX 24 kb)
