# Supplementary material for: Shape-Setting of Self-Expanding Nickel–Titanium Laser-Cut and Wire-Braided Stents to Introduce a Helical Ridge
Source: Cardiovasc Eng Technol. 2024 Feb 5;15(3):317–32. doi: 10.1007/s13239-024-00717-2 (PMC11239776; doi:10.1007/s13239-024-00717-2)
Supplement: Supplementary file 1 — Supplementary file1 (DOCX 24 kb) [file 13239_2024_717_MOESM1_ESM.docx]

Appendix

# Appendix - Crush Resistance Data

The results of crush test with parallel plates are reported in ***Table A 1*** in terms of average and standard deviation (SD). The test was repeated three times on each sample, computing the sample an average and standard deviation. Then, having an availability of three samples for each combination of heat treatment, the results were combined among samples, and standard deviations calculated using pooled estimators.

Table A 1 Crush load reported with average and standard deviation for the different heat treatment under study

| **Crush Load [N]** | | |
| --- | --- | --- |
|  | **Laser-cut**  [average ± SD] | **Braided**  [average ± SD] |
| **Heat Treatment** |  |  |
| Reference | 5.85 ± 0.06 | 13.17 ± 0.43 |
| 400°C/50 min | 4.37 ± 0.62 | 6.91 ± 0.26 |
| 450°C/50 min | 5.45 ± 0.58 | 8.30 ± 0.23 |
| 500°C/50 min | 7.11 ± 1.69 | 8.41 ± 0.25 |
| 500°C/40 min | 7.83 ± 0.37 | 7.60 ± 0.13 |
| 500°C/30 min | 7.67 ± 0.57 | 10.34 ± 0.16 |

# Appendix – Radial Compression Data

The evaluation of radial strength (*RS*), radial resistive force (*RRF*), chronic outward force (*COF*) and force at maximum crimping (*F*_max_) of crush test with parallel plates are reported in **Table A 2.**

Table A 2 Relevant force measurements with average and standard deviation for the different heat treatment under study

| **Radial Compression** | | | | | | | | |
| --- | --- | --- | --- | --- | --- | --- | --- | --- |
|  | **Laser-cut**  [average ± SD] | | | | **Braided**  [average ± SD] | | | |
| **Heat Treatment** | *RS*  [N·mm^-1^] | *RRF*  [N] | *COF*  [N] | *F*_max_  [N] | *RS*  [N·mm^-1^] | *RRF*  [N] | *COF*  [N] | *F*_max_  [N] |
| Reference | 52.78 ± 2.21 | 50.03 ± 1.65 | 30.37 ± 0.52 | 98.35 ± 3.00 | 10.95 ± 2.32 | 12.89 ± 2.02 | 3.65 ± 0.23 | 15.81 ± 0.52 |
| 400°C/50 min | 21.27 ± 0.49 | 19.58 ± 0.75 | 3.94 ± 0.23 | 77.32 ± 4.39 | 5.98 ± 1.84 | 8.10 ± 3.46 | 2.17 ± 0.41 | 15.56 ± 3.33 |
| 450°C/50 min | 23.50 ± 2.62 | 22.40 ± 3.59 | 6.71 ± 1.19 | 78.57 ± 1.67 | 9.12 ± 0.39 | 9.95 ± 1.42 | 2.60 ± 0.26 | 18.20 ± 0.68 |
| 500°C/50 min | 48.60 ± 3.66 | 47.31 ± 3.34 | 18.07 ± 1.71 | 95.87 ± 1.79 | 8.63 ± 0.77 | 9.47 ± 1.23 | 2.64 ± 0.12 | 20.75 ± 0.50 |
| 500°C/40 min | 51.96 ± 3.83 | 49.80 ± 3.73 | 21.03 ± 1.28 | 96.65 ± 2.54 | 9.72 ± 2.29 | 9.30 ± 2.62 | 2.54 ± 0.25 | 21.29 ± 1.76 |
| 500°C/30 min | 53.91 ± 0.14 | 51.55 ± 0.45 | 21.94 ± 0.40 | 100.52 ± 2.01 | 12.12 ± 2.75 | 12.31 ± 3.11 | 2.97 ± 0.32 | 23.00 ± 1.46 |

# Appendix – Transformation Temperature with DSC

Table A 3 Temperatures associated to the end of transformation to the austenite phase (A_f_)

|  | **Laser-cut** | **Braided** |
| --- | --- | --- |
| **Heat Treatment** | *A*_f_  [ᵒC] | *A*_f_  [ᵒC] |
| Reference | 24.33 ± 0.40 | - |
| 400 ᵒC/50 min | 45.77 ± 0.21 | 37.83 ± 0.65 |
| 450 ᵒC/50 min | 44.27 ± 1.67 | 36.73 ± 0.40 |
| 500 ᵒC/50 min | 31.20 ± 0.52 | 29.80 ± 0.40 |
| 500 ᵒC/40 min | 24.27 ± 0.76 | 25.80 ± 0.36 |
| 500 ᵒC/30 min | 23.90 ± 0.17 | 23.10 ± 1.25 |
